# Distorting anatomy to test MEG models and metrics

**DOI:** 10.1162/IMAG.a.1189

**Published:** 2026-03-30

**Authors:** José David López, Yael Balbastre, John Ashburner, Alberto Mariola, James J. Bonaiuto, Gareth Barnes

**Affiliations:** Engineering Faculty, Universidad de Antioquia UDEA, Medellín, Colombia; Global Brain Health Institute, Trinity College Dublin, Dublin, Ireland; Department of Imaging Neuroscience, UCL Institute of Neurology, University College London, London, United Kingdom; Department of Experimental Psychology, University College London, London, United Kingdom; Institut de Sciences Cognitives Marc Jeannerod, CNRS, UMR5229, Lyon, France; Université de Lyon, Université Claude Bernard Lyon 1, Lyon, France

**Keywords:** MEG/EEG brain imaging, diffeomorphic modeling, brain anatomy

## Abstract

The magnetoencephalographic and electroencephalographic (M/EEG) source reconstruction problem is an ill-posed model inversion, so it must be constrained by imposing biologically and physically plausible assumptions. Different M/EEG source reconstruction methods entail different assumptions about the underlying current distribution, yet all produce subjectively plausible current estimates. This work aims to develop an objective method that can be used to test any M/EEG analysis pathway. We make use of advances in diffeomorphic brain shape modeling to construct a set of parametrically deformable cortical surfaces that are representative of the population. These deformed (surrogate) brains provide a quantifiable parametric distortion from the ground-truth anatomy. If the current flow giving rise to the MEG data were generated on the true cortical manifold, MEG current estimates should be selective of the true anatomy. We show in simulation and with empirical data how the correct reconstruction assumptions depend closely on the true anatomy. We present a method to quantify the performance of MEG source reconstruction algorithms (and metrics of fit) in terms of millimeters of distortion.

## 1 Introduction

Magnetoencephalographic (MEG) and electroencephalographic (EEG) recordings enable reconstruction of spatio-temporal images of human brain function. Estimating these images from data measured outside the head requires additional assumptions, which are continually being refined, tested, and updated. We have proposed that anatomy (available from non-invasive Magnetic Resonance Imaging—MRI) provides a valuable ground truth against which to decide between assumption sets (Barnes et al., 2006; Little et al., 2018; López et al., 2017; Stevenson et al., 2014). We know that the primary current flow (which gives rise to the external magnetic fields) is bound (in the cell bodies of pyramidal neurons) to anatomy. We hypothesize that more accurate current flow models will be more likely given the correct, rather than incorrect, assumptions about anatomy.

The main issue addressed by this paper is the generation of realistic (brain-like) surrogate anatomy. Barnes et al. (2006) used rotated versions of the true anatomy within a spherical volume. Stevenson et al. (2014) and Little et al. (2018) progressively removed higher-order components from spherical harmonic approximations to the cortical manifold. While López et al. (2017) produced surrogate brains from linear mixtures of these harmonic components. All these cases, while providing proof of principle, set a relatively low bar on algorithm performance as the surrogate brains were not anatomically realistic.

In contrast, Troebinger et al. (2014) found higher evidence for generative models (of the MEG data) based on an individual’s true cortical anatomy, rather than another person’s brain warped into the same individual’s head. Here, we pursue this direction of using realistic surrogates but take advantage of recent advances in diffeomorphic brain shape modelling (Ashburner et al., 2019). This method was originally developed to enhance data mining of large MRI datasets, by parameterizing each structural MRI into a relatively small number of latent variables. Importantly, these latent variables, which describe a population based on large structural databases, are Gaussian. This means that it is possible to describe an individual’s brain shape as a vector of values and construct perturbations of this vector that distort this brain within realistic limits. The deformations here are subtler than one might expect when exchanging brains (in which patterns of gyrification may change), as the individual topology is preserved.

**Fig. 1.**
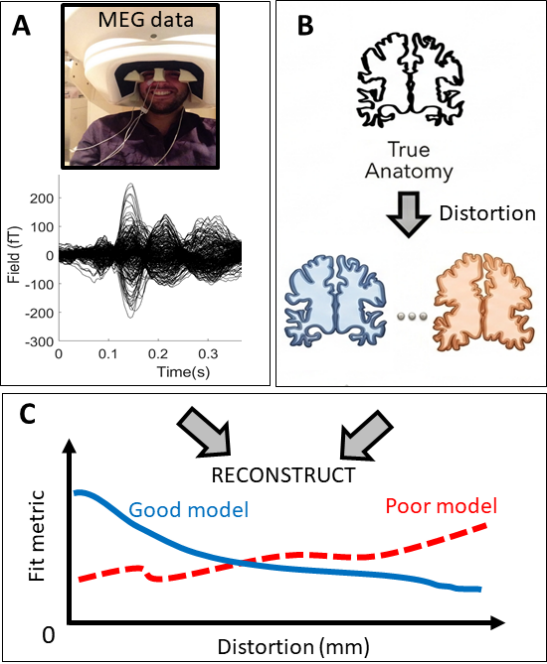
Overview of paper. (A) We take evoked response data from an MEG head-cast experiment. (B) These data are then reconstructed (under different modelling assumptions) onto the true, then progressively more distorted, versions of the subject’s cortical surface. (C) We plot a fit metric against distortion for each model. Models (and metrics) which reflect underlying brain function will be sensitive to the true anatomy; whereas poor models will not.

The methods can be summarized as follows (Fig. 1). We create surrogate cortices that deviate increasingly from the true anatomy. We then reconstruct MEG data onto these cortices using different algorithms (or models of brain function) and fit metrics. We describe a method to summarize any processing pipeline in terms of its sensitivity to the ground truth anatomy in terms of distortion (in mm). Algorithms that favor large distortions, or do not care onto which anatomy current flow is reconstructed, are unlikely to be useful.

## 2 Methods

We first describe the process of diffeomorphic modeling (or creating the surrogate brains). We then describe the parameter space we have chosen to work in, which involves stepping away from the true brain shape in different (pseudo-random) directions (in the high dimensional space).

### 2.1 Diffeomorphic modeling

The diffeomorphic algorithms used in this paper have been described extensively elsewhere (Balbastre et al., 2018). In summary, the shape of an individual’s brain, described by its tissue probability map, can be parameterized into 100 components each with a Gaussian distribution. Average brain shape is located at zero in this high-dimensional space and any individual’s brain shape can be described by a 100-component vector of z-scores. In this work, we extract the cortical mesh of an individual and then distort this mesh by perturbing it progressively through this space. A more comprehensive explanation follows.

Diffeomorphic shape modeling assumes that any individual anatomy can be described by a diffeomorphic (i.e., smooth and invertible) transform applied to a common template (Miller et al., 1993). Conversely, given a collection of N observed anatomies, an empirical template encoding the average anatomy of a population can be obtained by minimising its distance to all elements of the collection in a procedure known as geodesic shooting (Ashburner & Friston, 2011; Miller et al., 2006). In this framework, the diffeomorphism for subject *n* is encoded by an “initial velocity” ${\text{v}}_{n}\in {\mathbb{R}}^{3M}$ (where M is the number of voxels) and the distance between anatomies is penalized on the squared gradients (or some other differential operator $\text{D}\in {\mathbb{R}}^{F\times 3\;M}$ that describes smoothness) of this velocity field. After template construction, the posterior distribution of velocity fields can be efficiently estimated by performing a Bayesian residual component analysis (Bishop, 1999; Kalaitzis & Lawrence, 2011), a variant of principal component analysis that allows ‘residual’ variance (here, known smoothness) to be factored out of the projected variance when determining principal modes of variation. This procedure assumes that velocities can be factorized as ${\text{v}}_{n}=\text{W}{\text{z}}_{n}+\varepsilon_{n}$, where $\text{W}\in {\mathbb{R}}^{3M\times K}$ contains K≪3M principal components shared across individuals, ${\text{z}}_{n}\in {\mathbb{R}}^{K}$ is the latent projection of the individual onto the principal basis, and $\varepsilon_{n}$ is the residual vector with a covariance structure derived from the differential operator D. The model further assumes that the latent coordinates stem from a standard Gaussian, the residuals have the same covariance structure as the differential regularizer used during Geodesic shooting, and each principal component stems from a Gaussian with variance $\alpha_{k}$, which can be summarized as:

$${\text{z}}_{n}∼N\left(\text{0},\text{I}\right),{\text{w}}_{k}∼N\left(\text{0},\alpha_{k}\text{I}\right),{\text{v}}_{n}∼N\left(\text{W}{\text{z}}_{n},\sigma^{2}{\left({\text{D}}^{\text{T}}\text{D}\right)}^{-1}\right)$$ (1)

The model is fitted using variational Bayesian inference under the approximation that the posterior factorises across W and ${\text{z}}_{n}$, while the variances $\sigma^{2}$ and $\alpha_{k}$ are estimated by maximum-likelihood. This analysis allows each velocity field (and, therefore, each anatomy) to be described by a small number of latent projections (Ashburner et al., 2019; Balbastre et al., 2018; Zhang & Thomas Fletcher, 2015). New anatomies can then be encoded in this low-dimensional latent space by registering them with the template and projecting the resulting velocities onto the principal basis. Furthermore, random anatomies can be sampled from the learnt model by sampling latent codes from the standard Normal distribution.

Here, 662 structural MRI scans (with a 1:1 male/female ratio) were randomly sampled from four large datasets (“ABIDE”, n.d; “COBRE”, n.d; “IXI Dataset”, n.d; “OASIS”, n.d). They were segmented (Ashburner & Friston, 2005), resliced to 1.5 mm, and the grey and white matter tissues were diffeomorphically aligned with their common mean (Ashburner & Friston, 2011). A 100-component Bayesian residual model was fitted to the resulting velocity fields. Brains were rigidly aligned, meaning that brain size was encoded within at least one of these modes.

### 2.2 Parameter space

The shape model encodes variability using 100 eigenvectors to account for normal brain shape variation. Each individual therefore has a vector (${\text{z}}_{n}$) of 100 values that describe their brain shape and a corresponding cortical surface mesh. We then distorted this surface mesh by manipulating this individual vector. For clarity we now refer to the individual’s true ${\text{z}}_{n}$ prior to distortion as ${\text{z}}_{0}$; distorted versions of this original vector are ${\text{z}}_{\alpha }$. To avoid any confounds due to the volume of the mesh changing (i.e., sources becoming nearer to the sensors), we first modulated different ranges of components (e.g., 1–100, 4–100 etc) and selected a range (8–100) that maintained an approximately constant brain volume (Supplementary Fig. S1). We sampled each of these 93 components across a range of fixed points, which traversed a range of z-scores which modulated the distortion from the true brain in millimetres. A distortion trajectory is the defined by the random choices of -1 or +1 (denoted by $s_{i}$) at each of the components to be distorted (Eq. 2). For the lowest 7 components $s_{i}$ is set to zero to avoid distorting the brain size. The distortion at each component i then moves systematically from either {−Z to  +Z} or {+Z to  −Z} accordingly. The magnitude of this distortion (or point on the range between +Z and −Z ) is indexed by j which varies from 1 to N (Eq. 4). In this case, N=17 , Z=3 .

**Fig. 2.**
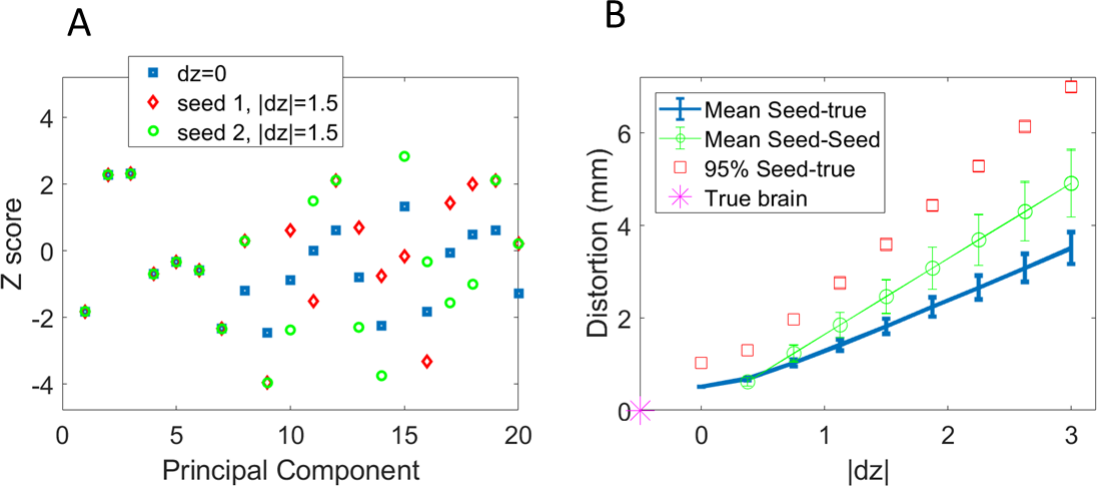
(A) Example of the first 20 (of 100) components defining the true cortical surface z (δz=0 , blue squares) for an individual. In addition, two trajectories (based on different random seeds) are shown for |δ z|=1.5 . Note that only components 8 and beyond are distorted. Each component is moved by |δ z| but the direction of movement is determined by the pseudo-random vector **s** (Eqs. 2 and 3). (B) Mean and standard deviation vertex-vertex distances between cortices on eight trajectories (eight pseudo-random vectors **s**) and the true surface (blue solid). The mean 95th percentiles of these distances are shown as red squares. Mean and standard deviation trajectory-trajectory (or seed-seed) vertex-vertex distances are shown as green circles. Note that at |δ z|=0 there is a non-zero distance to the true cortical surface. This is because the parameterization of the surfaces does not account for all spatial detail. An additional point (asterisk), at distance zero, has been added to represent the true (undistorted) cortical surface. Note that each trajectory consists of pairs of cortices at + |δ z| and - |δ z|. These cortices are distinct yet distorted by the same distance in mm from |δz|=0 . This means that 8 ((N-1)/2) pairs of distorted cortices overlap on this plot.

The random selection of direction per component (determined by the random seed that initializes the trajectory) means that it is possible to create several close-to-orthogonal trajectories for each subject (Supplementary Fig. S2). In other words, for each subject it is possible to create surrogate cortices that are distant to one another yet equally displaced (determined by j) from the true cortex (Fig. 2B).

For higher-order components, we define a random seed which gives a deterministic vector of random signs s, where:

$$s_{i}=2r-1,8\leq i\leq 100$$ (2)

where r is drawn from a random binary distribution P(r=1)=0.5, P(r=0)=0.5 , and $s_{i}$ is therefore (pseudo-randomly) either +1 or −1 .

For the lower-order components (controlling brain size), $s_{i}$ is set to zero.

$$s_{i}=0,0<i\leq 7$$ (3)

$$\delta_{z_{i,j}}=\frac{2Z\left(j-\frac{N+1}{2}\right)s_{i}}{N},1<i\leq 100,j\in \left\{1,2..N\right\}in\;\mathbb{Z}$$ (4)

For clarity of notation, let α comprise both the choice of a specific random seed (determining the vector s) and any point in the trajectory (indexed by j).

Then any distorted brain can be represented as

$${\text{z}}_{\alpha }={\text{z}}_{\text{0}}+\delta \;{\text{z}}_{\propto }$$ (5)

where ${\text{z}}_{\alpha }$ contains the eigenvalues describing a brain distorted by $\delta \;{\text{z}}_{\propto }$ from the original (${\text{z}}_{0}$).

### 2.3 Implementation

The diffeomorphic modeling was used to parameterize each subject’s MRI. The same MRI was used to extract the initial (ground-truth) individual white-matter cortical surface, constructed using FreeSurfer (Fischl, 2012). This mesh consisted of ~30,000 vertices.

This initial (FreeSurfer) surface was then deformed using warps above to generate an additional 17 surfaces (Eq. 4). This parameterized distortion had a direction (in the high-dimensional space) determined **s** (dependent on the choice of random seed). Each new surface gave rise to a new source model defining a set of current dipoles at each mesh vertex. The current dipole orientation was set to be normal to the cortical surface. The extra-cranial field due to this primary current flow, in turn, depends on the intervening media. Here, we used a single-shell (or corrected-sphere) forward model (Nolte, 2003) to provide a 10th-order multiple spherical harmonic approximation to the inner-skull boundary (see also Supplementary Figure S5 for single sphere model). That is, the exploration of parameter space is an exploration of different source models (each defined by a different surface mesh).

### 2.4 Framework overview

All the inversion is carried out within the statistical parametric mapping (SPM) Bayesian framework.

From the starting point that

$$\text{Y}=\;{\text{L}}_{\alpha }\text{J}+\varepsilon$$ (6)

Where $\text{Y}\in {\mathbb{R}}^{N_{c}\times N_{t}}$ are the measured MEG data with $N_{c}$ channels and $N_{t}$ time points. ${\text{L}}_{\alpha }\in {\mathbb{R}}^{N_{c}\times N_{v}}$ is the gain (or lead-field) matrix linking the $N_{v}$ source elements to all MEG channels for brain mesh α. The true underlying current density at each source over the $N_{t}$ time points is $\text{J}\in {\mathbb{R}}^{N_{v}\times N_{t}}$. The unmodeled noise is given by ε.

The most likely posterior estimate ${\hat{\text{J}}}_{\alpha }$ for the α-th surrogate brain is given by

$${\hat{\text{J}}}_{\alpha }={\text{Q}}_{\alpha }{\text{L}}_{\alpha }^{T}{\left({\text{L}}_{\alpha }{\text{Q}}_{\alpha }{\text{L}}_{\alpha }^{T}+{\text{Q}}_{\varepsilon }\right)}^{-1}\text{Y}$$ (7)

Where ${\hat{\text{J}}}_{\alpha }$ is the estimated current density, ${\text{Q}}_{\alpha }$ is the estimated source level covariance, and ${\text{Q}}_{\varepsilon }$ is the estimated sensor level noise covariance.

To provide a null in which the physical (lead-field) information was distorted, we switched ${\text{L}}_{\alpha }\;$ to ${\text{L}}_{\alpha ,null\;}\in {\mathbb{R}}^{N_{c}\times N_{v}}$ in which the $N_{c}$ rows are randomly interchanged. In this way, the data Y remains unchanged but any biophysical link to the true anatomy (determined by the lead fields) is broken:

$${\hat{\text{J}}}_{\alpha ,\text{null}}={\text{Q}}_{\alpha }{\text{L}}_{\alpha ,\;\;\text{null}}^{T}{\left({\text{L}}_{\alpha ,\;\text{null}}{\text{Q}}_{\alpha }{\text{L}}_{\alpha ,\;\text{null}}^{T}+{\text{Q}}_{\varepsilon }\right)}^{-1}\text{Y}$$ (8)

The underlying source distribution is estimated by maximizing the negative variational Free energy ($F_{\alpha }$) (Friston et al., 2007) (also known as the evidence lower bound or ELBO, https://en.wikipedia.org/wiki/Evidence_lower_bound):

$$\begin{array}{l} F_{\alpha }=-\frac{N_{t}}{2}\log\left|\Sigma_{\alpha }\right|-\frac{N_{t}}{2}\text{tr}\left(\Sigma_{Y}\Sigma_{\alpha }^{-1}\right)-\frac{N_{t}N_{c}\log\left(2\pi \right)}{2}\\ \;\;\;\;\;\;\;\;\;\;\;\;-\frac{{\left(\hat{\lambda }-\text{v}\right)}^{T}\Pi \left(\hat{\lambda }-\text{v}\right)}{2}-\frac{\log\left|\Sigma_{\lambda }\Pi \right|}{2} \end{array}$$ (9)

Where |⋅| is the matrix determinant operator, *“tr” (⋅)* is the trace operator, $\Sigma_{Y}=\frac{\text{Y}{\text{Y}}^{T}}{N_{t}}$ is the data covariance, and $\Sigma_{\alpha }=\left({\text{L}}_{\alpha }{\text{Q}}_{\alpha }{\text{L}}_{\alpha }^{T}+{\text{Q}}_{\varepsilon }\right)\;$ is the posterior model covariance at sensor level. The hyperparameters λ are assumed to have Gaussian prior and posterior distributions: $q_{0}\;\left(\lambda \right)=N(\lambda ;\text{v}$ , $\Pi^{-1}$ ) and $q\left(\lambda \right)=N(\lambda ;\hat{\lambda },\Sigma_{\lambda }$) respectively (Friston et al., 2008).

The key insight to this equation is in the first (sparsifying) term which penalizes the solution based on the determinant of the modeled data covariance. Namely, if the model covariance has a large volume (a high rank, or more constituent sources), it is penalized (Wipf & Nagarajan, 2009).

### 2.5 Inversion schemes

We use common inversion algorithms to explain the measured MEG data as current flow constrained to each surface on the trajectory in turn. Each algorithm can be defined by the modeling assumptions that determine the covariance of neuronal current flow across the cortex (López et al., 2014; Mosher et al., 2003). The models tested were all implemented in the open-source software package SPM (Litvak et al., 2011; Tierney et al., 2025), and were:

- **IID**: Minimum Norm algorithm (Hämäläinen et al., 1993). In SPM, this is implemented as single diagonal source variance component and a diagonal sensor level noise variance component which are then weighted (based on the free energy optimization above) in order to regularize the solution (Friston et al., 2008; López et al., 2014).
- **GS**: Greedy Search Algorithm—enforcing sparseness (Friston et al., 2008; López et al., 2014). In SPM, this is implemented in terms of multiple pre-defined patches of cortex (or priors) which are optimally mixed to explain the measured data outside the skull.
- **EBB**: Empirical Bayes Beamformer. In this case, a single prior is defined using beamformer assumptions (unit gain, no zero-lag covariant sources etc.) (Van Veen et al., 1997). This prior is then mixed with a diagonal estimate of sensor noise (as in the IID case) (Belardinelli et al., 2012).

In this work, we look at three additional metrics through which to quantify an inversion scheme.

- **Variance explained** (i.e., the squared correlation coefficient between measured and predicted data):
$$R^{2}=\frac{\text{SST}-\text{SSR}}{\text{SST}}$$ (10)
  where
$$\text{SSR}=||Y-LJ|{|}_{F}^{2}\;\text{and}\;\text{SST}=||Y|{|}_{F}^{2}$$ (11)
  Where $||..|{|}_{F}$ denotes the Frobenius norm.
- **Cross-validation error** (Bonaiuto, Rossiter, et al., 2018; Browne, 2000; Little et al., 2018). Here, we compute cross-validation error as the average root mean square (RMS) difference in femto-Tesla (over the 10 cross-validation runs) between the predicted and measured values in the 10% of MEG channels ($N_{c}^{'}$ below) excluded from the source reconstruction (and randomly selected in each cross-validation run). For example:
$$CV=\frac{1}{{N^{\prime }}_{c}}\sum_{1}^{{N^{\prime }}_{c}}\sqrt{\frac{1}{N_{t}}\sum_{1}^{N_{t}}{\left(y_{i,j}-{\hat{y}}_{i,j}\right)}^{2}}$$ (12)
- **Maximum normalized projected source power**. This metric is based on the observation that power-estimates (normalized by noise) are maximized for correct source models (Vrba, 2002). This works for classical LCMV beamformer estimates in which there is no explicit optimization and had been used in several studies of this nature (Barnes et al., 2006; Hillebrand & Barnes, 2003, 2011). For each source, the normalized projected power is given by the weights (which map the sensor data to source location θ), the data covariance $\Sigma_{\text{Y}}$ and normalized by the power projected by the sensor noise $\Sigma_{\varepsilon }$.
$$\max\left|\Gamma_{\theta }=\frac{W_{\theta }^{T}\Sigma_{Y}W_{\theta }}{W_{\theta }^{T}\Sigma_{\varepsilon }W_{\theta }}\right|$$ (13)
  where θ covers the range of source locations and orientation. This metric does not appear in the main manuscript but is used in Supplementary Figure S7.

### 2.6 MEG simulations

All simulations were run within the current (2026) SPM release (Tierney et al., 2025). The shared code (*spm_sim_example_distort_mesh.m*), based on simulating a single source in the motor cortex of the canonical subject, can be used to create Figure 4. The source consisted of 336 trials of a single dipole (with no spatial extent) simulated at MNI coordinates [x = -40, y = -18, z = 48] and a single-trial sensor-level SNR ≈ -1.77 dB and -17.43 dB at peak and median sensors respectively.

See also Supplementary Figure S7 which shows the generalizability of the method using common algorithms (sLORETA, eLORETA, LCMV) with/without orientation constraints (*spm_sim_example_distort_mesh_daiss.m*) and the maximal normalized projected source power metric (Barnes et al., 2006; Hillebrand & Barnes, 2003, 2011; Vrba, 2002) as a cost function.

### 2.7 MEG empirical data

We used previously published data from MEG recordings of eight subjects using head-casts (Bonaiuto, Meyer, et al., 2018; Little et al., 2018) (see data-availability section for an updated link). The head-cast minimizes subject movement during the experiment and gives a precise estimate of the relative positions of the sensor array and head (Meyer et al., 2017; Troebinger et al., 2014). In brief, recordings were made using a 275-channel CTF Omega MEG system. The data were sampled at 1200 Hz, filtered (5th-order Butterworth band-pass filter: 2–100 Hz, Notch filter: 50 Hz), and down-sampled to 250 Hz. Eye-blink artefacts were removed using multiple source eye correction (Berg & Scherg, 1994). Participants completed a visually cued action decision-making task in which they responded to a Random Dot Kinematogram (RDK) (displayed for 2 s with coherent motion, either to the left or right). Following a 500 ms delay, an instruction cue appeared, pointing either to the left or the right, and participants were instructed to press the corresponding button (left or right) as quickly and as accurately as possible.

The processing of the data was either focused on trials locked to the onset of the instruction cue (0 to 500 ms), the random dots (-2500 to -2000 ms) or to the button press (-200 to 300 ms). Time zero and epoching were as defined in Bonaiuto, Meyer, et al. (2018). These trials were first averaged and filtered to a 5–90 Hz bandwidth.

## 3 Results

We now move through the space of possible cortical surfaces and examine the impact on MEG reconstruction algorithms and fit metrics. We first show how the cortex is progressively deformed from the anatomical ground truth. We show the effect of this distortion on the reconstruction of simulated data. We go on to test whether this deformation has any influence on the metric or method of source reconstruction for real data (recorded using a head-cast). The logic is that distortions from the true anatomy will be of little consequence for sub-optimal metrics or reconstruction assumptions.

### 3.1 Deformations

Figure 2A shows two potential distortion trajectories (Eqs. 2 to 5). The shape of any brain in the population can be defined by a 100-element vector $z_{0}$ (blue squares). We then define a pseudo-random deformation trajectory (Eqs. 2 and 3) to move each person’s cortical mesh about $z_{0}$ in 17 successive steps (Eqs. 4 and 5). Cortical surfaces 1 and 17 are at the extremes of the trajectory, with surface 9 at the origin, where δz=0 (i.e., closest to the original cortical surface). Figure 2B shows the average distance of each vertex to the corresponding vertex on the original cortical surface (distributions shown in Supplementary Fig. S3). The true cortical surface (asterisk) on the x-axis is added for reference (as the trajectories do not capture all of the anatomical variance). Note that surfaces 1 and 17 and all pairs (2,16; 3,15 etc.), which have equal (but opposite) distortion of $z_{0}$**,** overlap on this plot*.* Also shown in Figure 2B are the distances between surfaces lying on different trajectories (determined by the pseudo-random vector s) and one another. That is, even though the trajectories are all a similar distance from the true brain, they are also distant from one another. Supplementary Figure S2 shows the correlation (mean r^2^ = 0.046) between the vectors δ z that define each trajectory.

**Fig. 3.**
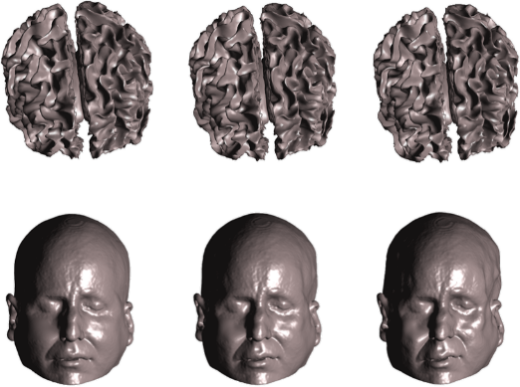
Top panel. Cortical meshes from one example trajectory at points |δ z|=0 , |δ z|=1.5 and |δ z|=3 (left-right respectively), which show increasing amounts of distortion. Figure 4A shows a superposition of coronal slices in which the difference between surfaces is more apparent. For illustration only, the lower panel shows the (70% reduced in size) scalp surface of this subject distorted in the same way. Note the gradual distortion of the face moving from left (truth) to right (distorted).

**Fig. 4.**
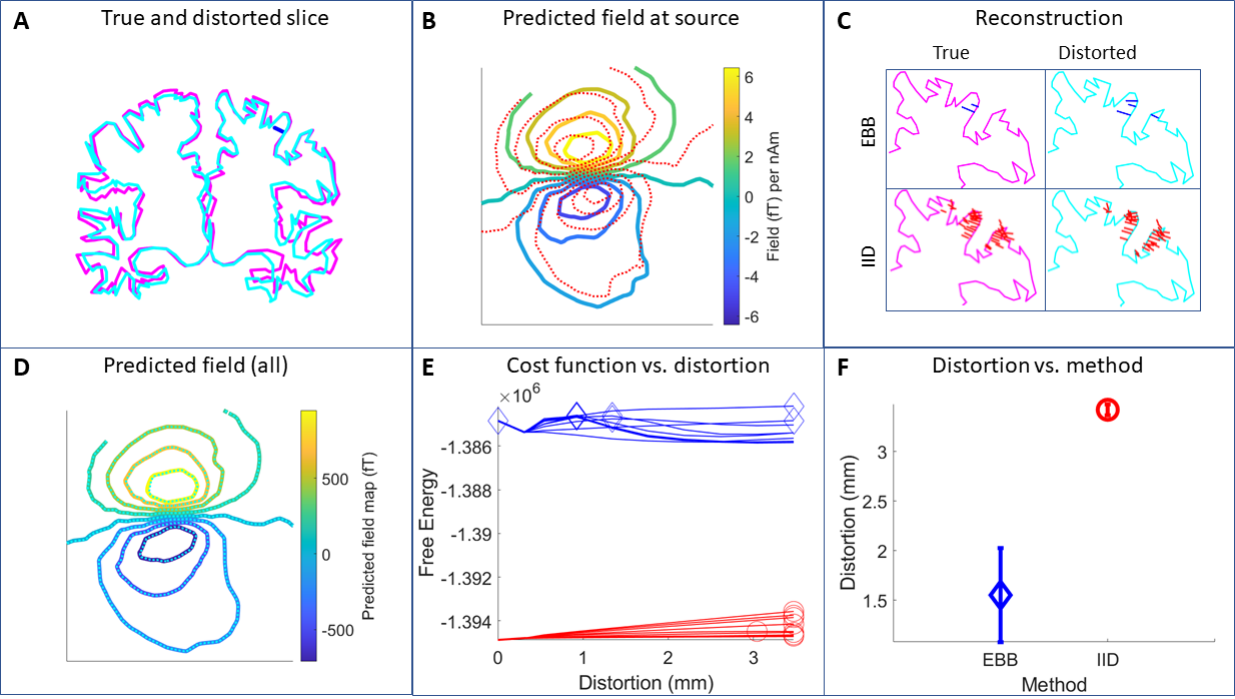
(A) Simulated source (dark blue bar) on a coronal slice through the true cortical mesh (magenta). Also shown is a cortical mesh (thin light blue lines) at the extreme of the trajectory (|δ z| = 3) with mean vertex-vertex distortion from original of 3.5 mm. (B) The magnetic field profile at sensor level due to the original source (colors) and alongside (dotted) the magnetic field pattern due to the same current element on the distorted surface. (C) The source reconstruction under EBB (top) and IID (lower) assumptions onto the undistorted (left) and maximally distorted cortices (right). (D) The field-maps predicted by all current estimates in C give rise to sensor-level field maps that explain 99% of the variance of one another. Shown are the field-maps due to IID reconstructions (lower panel C) of the same data onto distorted (dotted) and undistorted cortices. The field-maps explain 99% of the variance of one another. The same is the case for EBB (99% of IID field map explained) (E) The free energy associated with reconstructions onto the different cortices as distortion is increased for eight different trajectories and two different algorithms (EBB, IID). Diamonds and circles mark the peak metric (signifying most likely underlying cortex) for EBB and IID respectively. (F) Each peak (in E) gives a distortion. Taking the mean distortions across the eight trajectories gives a single distance measure for each algorithm.

Figure 3 shows three points on one trajectory of brain deformations, alongside similarly deformed scalp surfaces. As it is difficult to spot these cortical deformations by eye, the subject’s scalp surface is shown below to illustrate the deformation as |δ z| increases in magnitude. Note the gradual distortion of the face moving from left (truth) to right (distortion of lips and face). Coronal slices through the original (top left) and deformed (top right) surfaces are shown in Figure 4A.

### 3.2 Simulated data

We first show an example of this methodology with simulated data. A single source was placed in the motor cortex based on the true brain shape (i.e., true orientation and position of this source). The simulated data were reconstructed onto brains moving across eight separate distortion trajectories. Reconstructions were based either on IID (distributed) or EBB (sparse) assumptions. We show the goodness of fit using Free-Energy in this case.

Figure 4 provides an example in simulation (*spm_sim_example_distort_mesh.m* creates this). A single source is simulated on the cortical surface (Fig. 4A). It is reconstructed with matching and ill-matching assumption sets (in this case EBB and IID algorithms respectively) onto progressively more distorted surfaces. A coronal slice through the true cortex and a distorted cortex at |δ z|=3 are shown in Figure 4A. The site of the original current element is moved (in orientation and position) such that its impact on the measurement channels has changed (Fig. 4B). Figure 4C shows these data reconstructed under EBB (top) and minimum norm assumptions (lower) onto the true (left) and distorted cortices (right). The distortion, however, has no noticeable impact on the estimated field at the sensor level between algorithms or between distortions (Fig. 4D). In Figure 4E the Free energy cost function is used to quantify the solutions as distortion is increased for eight different distortion trajectories. The peak of the cost function (or best anatomical model) in each case is marked (diamonds for EBB, circles for IID). For the IID (incorrect assumptions) note that the cost-function increases (model becomes more likely) as the underlying anatomy becomes more distorted. For EBB (correct assumptions) however, the best models tend to peak closer to zero distortion. Figure 4F shows that the estimated distortion, in millimeters, can be used to judge between inversion schemes.

### 3.3 Real data

We now apply the same approach to real data (Bonaiuto, Meyer, et al., 2018). These data were recorded using subject-specific head-casts so as to minimize co-registration error. In an ideal scenario, the model fit metric should improve for brain structures closer to reality. We split this section into a comparison between algorithms, and then between metrics of fit.

### 3.3.1 Comparing algorithms

**Fig. 5.**
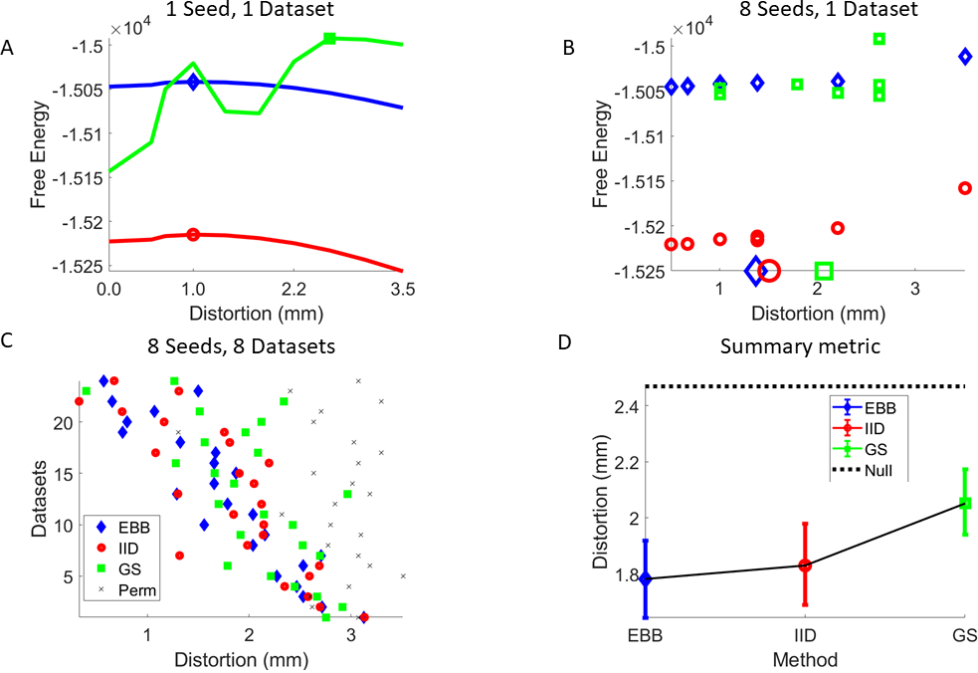
Empirical head-cast data. Inverted using three different algorithms EBB (blue diamonds), IID (red circles), and GS (green squares). (A) Free Energy (one trajectory, 1 dataset) as the cortex is distorted from 0 to 3.5 mm (mean vertex-to-vertex distance). The peaks (marked) indicate which cortices are most likely to support the measured data. Note that, by the Free-Energy metric alone, the most-likely anatomy is found by the GS algorithm at a mean distortion of ~3 mm. (B) Peak locations for 8 trajectories for the same dataset for three different algorithms EBB (blue diamonds), IID (red circles), and GS (green squares). The mean distortions estimated for each algorithm in this dataset (large markers on x-axis) are taken to the next level of inference. (C). Estimated distortions for the three algorithms over eight subjects and three datasets per subject. Distortions are ordered (top to bottom) in terms of increasing mean distortion (for visualization purposes). The crosses show the distortions estimated using the null models for the EBB algorithm (D) One can then quantify algorithm performance in terms of the mean distortions (in **C**) in millimetres. All algorithms perform better than chance (p < 0.05, black-dotted, set by the null models). EBB significantly (p < 0.05) outperforms GS.

Figure 5A shows the negative variational Free Energy for EBB, IID, and GS algorithms for one trajectory and a single dataset. The peaks are marked. Note that the best model (as quantified purely by Free Energy) is the GS algorithm and a brain distorted by 3 mm. Note also that IID and EBB algorithms favor brains closer to the ground truth (0 distortion). The original averaged data, source level images, Free energy values, and time-series for the EBB case (distorted and undistorted) are shown in Supplementary Figure S4. This process is now repeated across 8 trajectories and the peak fit metric (in mm of distortion) noted (Fig. 5B). For each algorithm the mean peak location (in mm) (large symbols) is taken to the next level of analysis. Figure 5C shows the empirical data for eight subjects and three datasets per subject. The distortions (per dataset) are ordered according to how close they are to zero in order to aid comparison. In addition, the null model (Eq. 8), using the EBB algorithm, is shown in black crosses. Figure 5D shows the mean and standard errors of the distances between the true brain and the algorithm-preferred brains. All algorithms come closer to the true brain than would be expected by chance (p < 0.05) (dotted, and set by the null models). In this case, the only significant difference (between algorithms, one-tailed t-test) is that EBB improves over GS (t(23) = -1.89, p < 0.036).

### 3.3.2 Comparing metrics

**Fig. 6.**
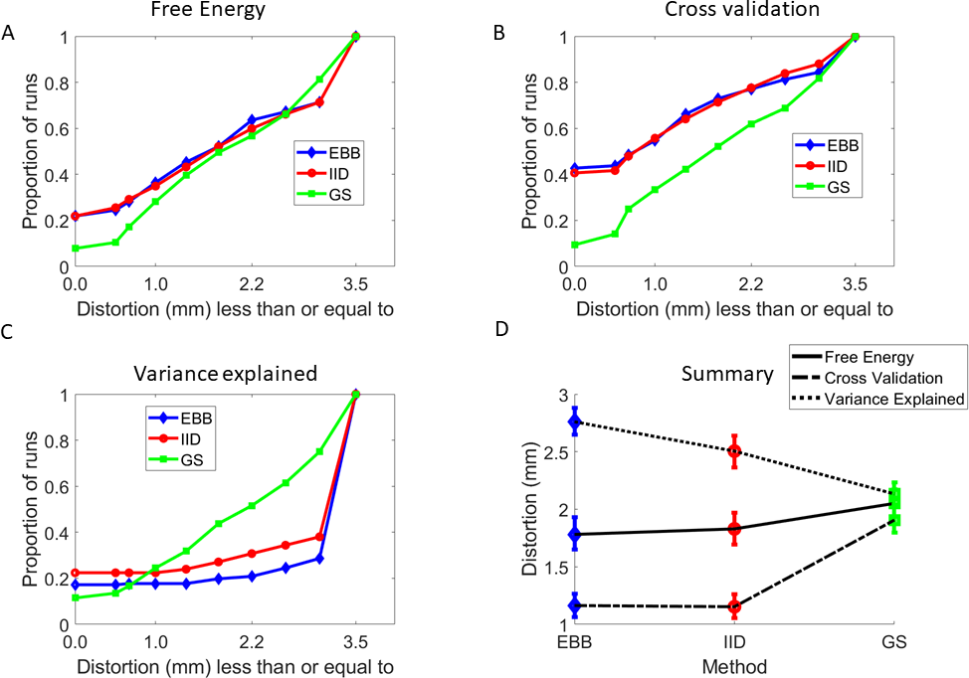
(A–C) Comparing metrics and algorithms EBB (blue triangles), IID (red circles), GS (green squares). (A) The Free Energy metric and the three different algorithms; (B) The cross-validation metric; (C) the variance explained. (D) Summary figure showing mean distortions for the three algorithms scored using the three different metrics: Free-energy (solid line), Cross-validation (dashed line), and variance explained (dotted line).

It is possible to run the same analysis but using different combinations of fit algorithm and metric. Figure 6A shows the data from Figure 5 plotted as a function of the number of datasets showing less than a certain distortion using the metric of Free Energy. Larger values to the left of the curve (or larger area under curve) indicate better performance. Figure 6B scores the same algorithms using cross-validation. In this case, note that in 40% of the datasets the true brain (zero distortion) is found. Figure 6C shows the algorithms scored according to variance explained. Note the relatively poor performance of IID and EBB algorithms under this metric. Summarizing in terms of mean distortion, it is clear that (for these data) cross-validation (dashed line) arrives at closest to the ground truth for EBB and IID algorithms. In this case, one-tailed t-tests show significant difference for EBB vs. GS (t(23) = -5.49, p < 0.0001) and IID vs. GS (t(23) = -5.50, p < 0.0001).

These comparisons are not limited between algorithms or metrics. Supplementary Figure S5 shows that alternative forward models (in this case the single sphere) can also be compared.

## 4 Discussion

We leverage the knowledge of true cortical anatomy to compare among MEG analysis pathways using a metric of millimetres. We have done this using realistic and quantifiable deformations to distort an individual’s cortical surface. We first show this in simulation (Fig. 4). We then show that the same applies for real-MEG data. All algorithms tested significantly (p < 0.05) outperformed chance behavior as defined by the null case (Fig. 5D). We found that inversion schemes based on the assumption sets behind IID and EBB were most selective of the true anatomy; and that this selectivity was improved when using a cross-validation metric (Fig. 6D).

This approach could be used to empirically determine best practice at any stage of the MEG (or EEG) pathway from the choice of forward models to sensor type. Importantly the metric of performance is in terms of millimetre distortion from true anatomy.

We should note that these findings are not restricted to a Bayesian framework. One can select other metrics such as peak normalized projected power (Barnes et al., 2006; Hillebrand & Barnes, 2003, 2011). Several commonly used algorithms (including LCMV (Van Veen et al., 1997), eLORETA (Pascual-Marqui et al., 2011), sLORETA (Pascual-Marqui, 2002)) are made use of in the demo-code (spm_sim_example_distort_daiss.m). Supplementary Figure S7 shows the sensitivity of these algorithms to cortical distortion with and without orientation constraints. As one might expect, the use of the cortical orientation makes all algorithms more sensitive to changes in the underlying anatomy.

It is interesting to note the contrast between the simulated (Fig. 4) and real data (Figs. 5 and 6). In simulation, there was a very clear difference between IID and EBB models. In the real data, however, there was little difference. There could be a number of reasons for this: i) The activity was primarily cortical and superficial and so it could be more difficult to distinguish between IID (typically superficially biased) and EBB estimates. ii) The EBB is not a classic beamformer algorithm (Van Veen et al., 1997) but rather uses an empirical beamformer prior as part of a generative model (Belardinelli et al., 2012). In cases in which a conventional beamformer might fail (for example zero lag correlated and distinct sources) there is no useful information in the prior and it becomes flat—defaulting to minimum norm behavior. In other words, EBB and IID will tend to the same algorithm in certain cases. iii) Finally, and perhaps most-likely, neither the assumptions between EBB or IID generative models match the true neuronal generative model and/or algorithm performance is constrained by some other factor (for example, sub-optimal volume conductor model, tissue conductivity, or segmentation errors).

An alternative method of validation is to see how the distortion metric performs when one subject’s data is used to estimate the best fitting anatomy from across the group. Supplementary Figure S6 shows that (for EBB) the most likely anatomy matches the MEG data it corresponds to for 6 of 8 subjects (p < 0.0001).

We used the white matter (rather than pial) surface and made no attempt to optimise the orientation of current flow (Bonaiuto et al., 2020). We used exclusively cortical surfaces; but if the manifold on which sub-cortical sources sit is well defined (Attal & Schwartz, 2013; Meyer, Rossiter, et al., 2017) the same approach could be used. Likewise, rather than distort the cortical surface, the same code can be used to also distort any of the conductivity boundaries (such as the inner skull).

In the limit of infinite data, all estimates should converge toward the true anatomy. Using longer-recordings and novel data processing techniques (e.g., Hidden Markov Models—Woolrich et al., 2013) would allow one to make use of all data recorded (Little et al., 2018; Martinez-Vargas et al., 2016).

### 4.1 Limitations

We have made use of the Nolte Single Compartment (or Single Shell) model which has been shown to outperform single shell BEMs but not more complex models with more compartments (Stenroos et al., 2014). We also made use of the single sphere forward model (Supplementary Fig. S5). There were two motivations for this. Firstly, to test a model that does not depend on the inner skull-boundary (which may be crossed during distortion) but simply requires specification of the volume centre (Sarvas, 1987). Secondly, to show how the method could be generalized (to more complex forward models in future). On the positive side, the findings between the two models were similar—indicating that our results are not due to crossing the inner-skull boundary. Surprisingly however, the simpler sphere model outperformed the corrected-sphere model (Supplementary Fig. S5). Future work, using more complex forward models, could examine the role that changes in CSF and white-matter compartments have on the solution. The main computational drawback is that a new set of lead-fields must be computed for each distortion and seed.

It should be noted that the implementation of GS used here is most likely sub-optimal in that only a fixed number of discrete (pre-defined) cortical patches are allowed to be active. Previous work has combined the greedy-search with an automatic relevance determination (ARD) stage (Friston et al., 2008): the greedy search to find variance components (source distributions) that maximize the model evidence and ARD to prune away those that make least contribution to the evidence. Alternatively, more computationally intensive approaches have been suggested in which model evidence is assessed over multiple possible patch locations to overcome problems with sparse sampling; and this, in turn, is run over multiple iterations to avoid local maxima (Lopez et al., 2012).

The averaged evoked data shown are specific to one experiment derived from three time-windows and in a single frequency band over eight subjects. We cannot, therefore, generalize findings across other datasets.

The cortical deformations are based on eight (random) distortion trajectories for all subjects. In order to observe any change in how the measured MEG data can be expressed on the cortical surface, we rely on the distortion perturbing cortical sources that will influence the estimate of current flow. However, distortion trajectories must exist that minimally influence this estimate; in which case we would see no change in model fit with distortion.

### 4.2 Perspective

Note that these data are based on head-cast recordings. Here, subjects wore individualized foam helmets that fit the scalp internally and the MEG system externally. That is, the subjects’ heads were immobilised in the scanner. Therefore, the relative location of the subject’s anatomy and the sensors is well-defined and fixed. This scanning method is time-intensive, only appropriate for healthy compliant subjects, and carries a certain amount of risk (due to the potential of MEG system movement whilst the subject’s head is fixed). We used head-cast data here as for this method the knowledge of true anatomy is essential. However, in practice, robustness to (inevitable) co-registration errors in an inversion algorithm is desirable. We suggest that this constitutes an initial stage to establish the relative performance of an algorithm (or metric). Once these algorithms (or assumptions about source covariance) have been established, the anatomical precision is no longer required.

Co-registration may also become less of an issue with a new generation of MEG sensors known as optically pumped magnetometers (OPMs) (Tierney et al., 2019), which can be worn for long periods, and their location relative to the underlying anatomy can be well defined. Although head-cast recording may be limited, OPMs provide an exciting and expanding field to test these methods further. This is important as OPMs allow subjects to perform tasks (like dancing (O’Neill et al., 2025)) which expand the literature yet cannot be replicated in many fixed scanning systems. In the limit of increasing measured data for an individual, these tests between competing anatomies should converge to a single solution (the true individual anatomy). The more data available per-subject, the more constrained (and well-posed) the problem becomes.

## Supplementary Material

Supplementary Material

## Data Availability

The code is part of the current development version of SPM (https://github.com/spm/spm). The deformation templates will be automatically downloaded the first time the algorithm is used. The code to train the Bayesian Residual Component Analysis model is available at https://github.com/balbasty/BRCA. The MEG-evoked response data, individual MRIs, and cortical surfaces are available here: https://figshare.com/s/e3ff829753a76bcfda22. The additional code to reproduce the simulations and single-subject analyses can be found at https://github.com/barnesgr123/spm_distort/ and within the latest version of SPM. The complete dataset, originally from Bonaiuto, Meyer, et al. (2018) and Little et al. (2018), can be found at https://osf.io/eu6nx.
