# Supplementary material for: Distorting anatomy to test MEG models and metrics
Source: Imaging Neurosci (Camb). 2026 Mar 30;4:IMAG.a.1189. doi: 10.1162/IMAG.a.1189 (PMC13037658; doi:10.1162/IMAG.a.1189)
Supplement: Supplementary Material [file IMAG.a.1189_supp.pdf]

Supplementary information for

Distorting anatomy to test MEG models and metrics

Lopez et al.

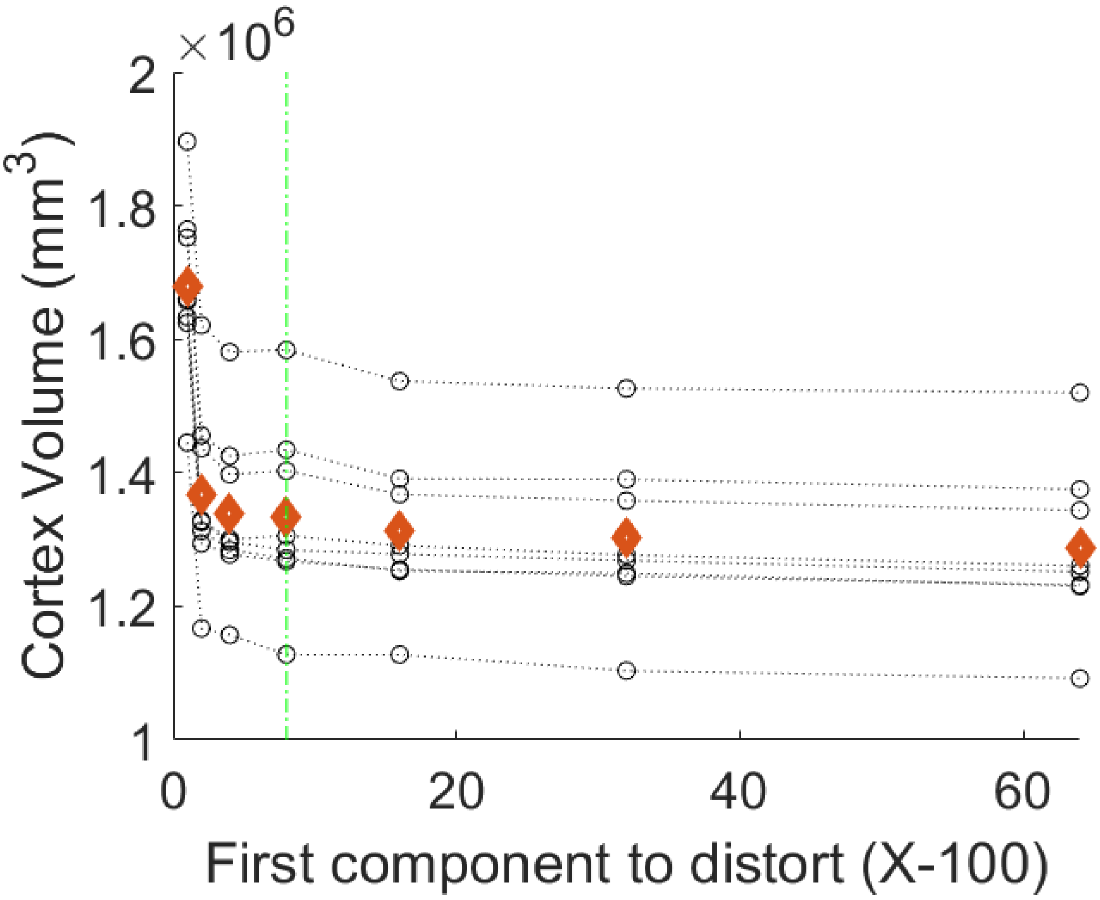

Figure S1. *Change in volume of cortex as different components (X-100) are manipulated. Each subject shown by circles and thin dotted line. Group average volume shown by diamonds. Components 1-100 (far left) give rise to greatest volume change. Volume change begins to plateau around range 8-100 (green dotted line).*

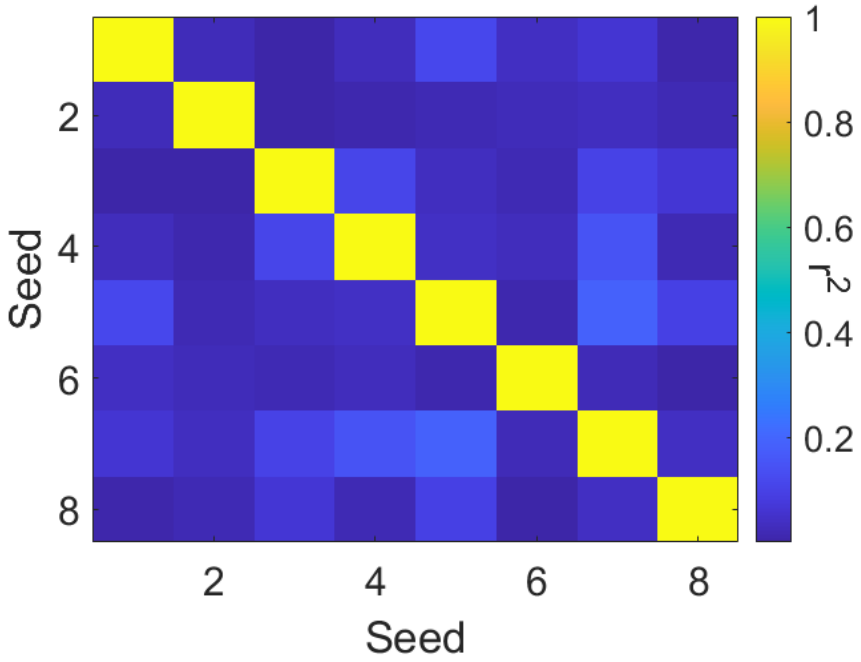

Figure S2. *Squared correlation coefficients (colour-scale) between the pseudo-random vector* ***s*** *(determined by the choice of initial random seed) used to make 8 different distortion trajectories for one subject. The distortions are orthogonal yet distort the cortex by a similar amount (figure 1B, main manuscript).*

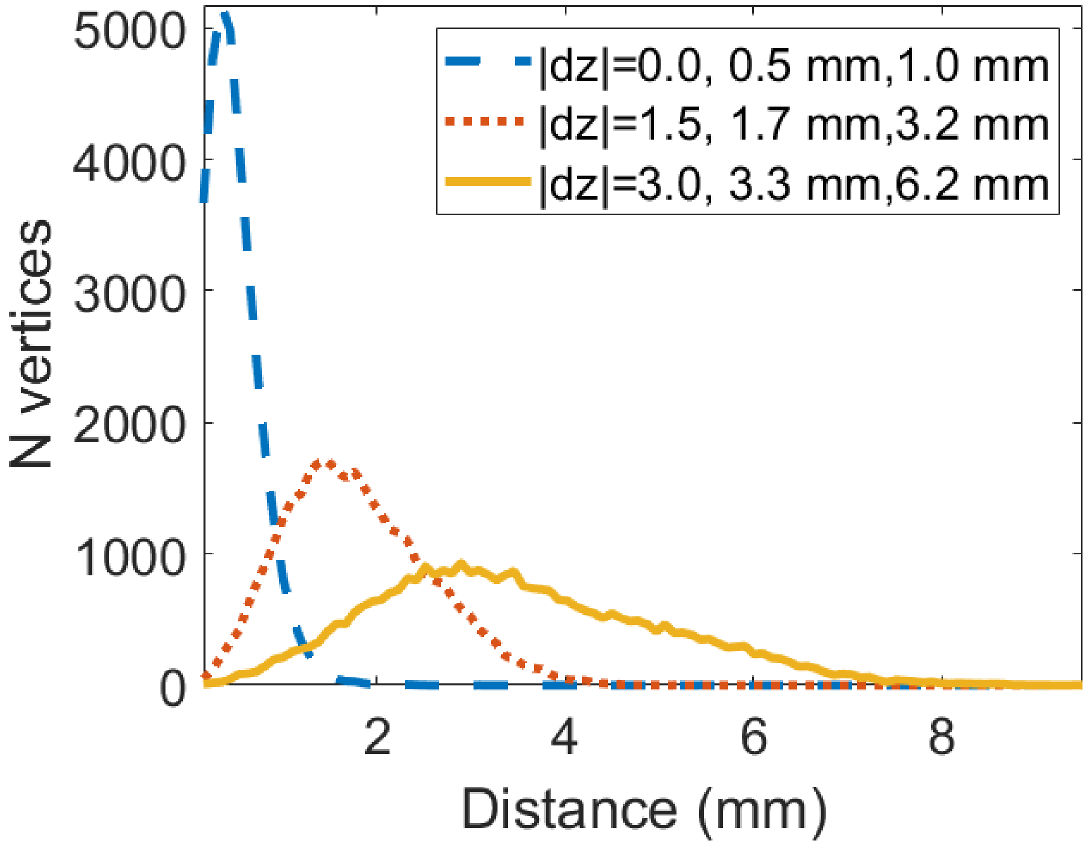

Figure S3. *Distribution of original vertex- perturbed vertex distances that make up a trajectory. Different curves represent different points on a single trajectory (blue dashed |dz|=0; red dotted |dz|=1.5; solid yellow |dz|=3.0). Legend shows |dz| value, mean distortion and 95th percentile distortion. For example, trajectory point |dz|=3 corresponds to a mean (vertex-vertex) distortion of 3.3mm; in which 95% of vertex-vertex distances are less than 6.2mm. Note that at |dz|=0 there is ~0.5mm mean distortion from the truth.*

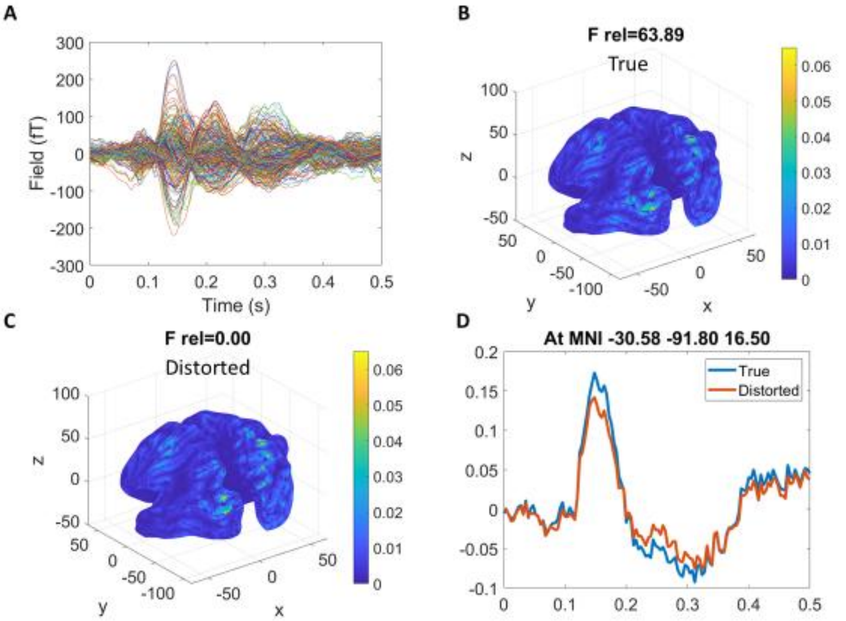

Figure S4. Averaged *response from first task (visual evoked response to instruction cue).**A**. Sensor level data over time (different channels different colours). **B**. R.M.S. Current density image on inflated MNI brain of these data (in A) source reconstructed onto original (true) cortical surface using EBB algorithm. **C**. Current density image on inflated MNI brain of r.m.s. current density. Note relative Free-Energy is lower (log units) than free energy observed in B. **D**. Current density estimates over time at peak vertex (from sum of B and C) for reconstructions onto true (blue) and distorted (red) cortices. This figure is created as part of the output of spm_distort_mesh_example.m .*

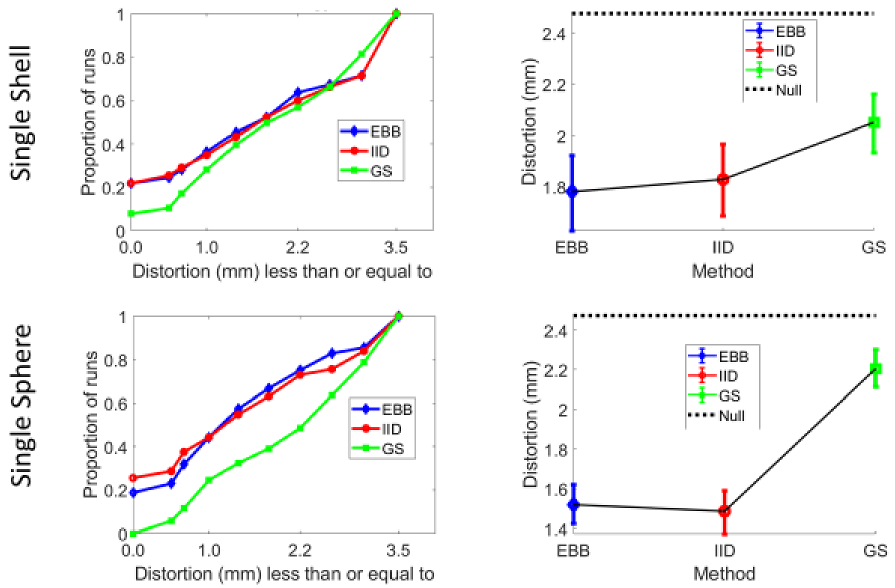

Figure S5. *Comparing single-sphere (top) and single-shell (lower) forward models. Left panels show the proportion of datasets with distortions less than or equal to a given value. Better models should have larger values at low distortion. Right panels show mean distortions for the 3 algorithms used. Note the y-axes. Performance improves significantly (when switching from single-shell to single sphere) for EBB, t(23)=2.07,p<0.05; IID, t(23)=3.56, p<0.002; with no change in GS, t(23)=-1.03,p<0.31.*

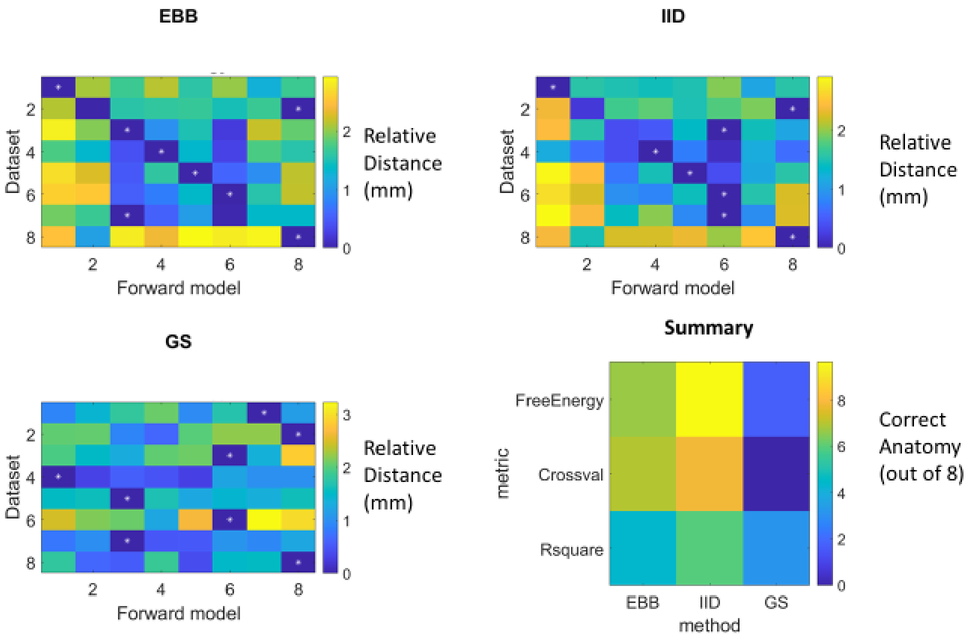

Figure S6. *Computing the distance metric but based on alternative anatomies (forward models). For example, the top row shows estimated distortion when the MEG data from subject 1 is paired with the brains/anatomies of all subjects. Relative distance means that the minimum distance (over forward models) has been subtracted (i.e. closest forward model is at zero). The white star indicates the minimum distance for each dataset. Ideally these stars would like along the diagonal (each forward model best matches each dataset). Three algorithms are shown (EBB, IID, GS) in which distance is judged using the free energy metric. The summary figure (lower right) shows the number of correct forward models identified (i.e. number of stars along the diagonal) for different combinations of algorithm and fit metric. Based on Binomial distribution p(3 or more correct)=0.018, p(4 or more correct)=0.0025, p(5 or more correct)=0.0002, p(6 or more correct)=0.00001. Note that these data only derive from the first dataset (i.e. 1/3 of data used in paper).*

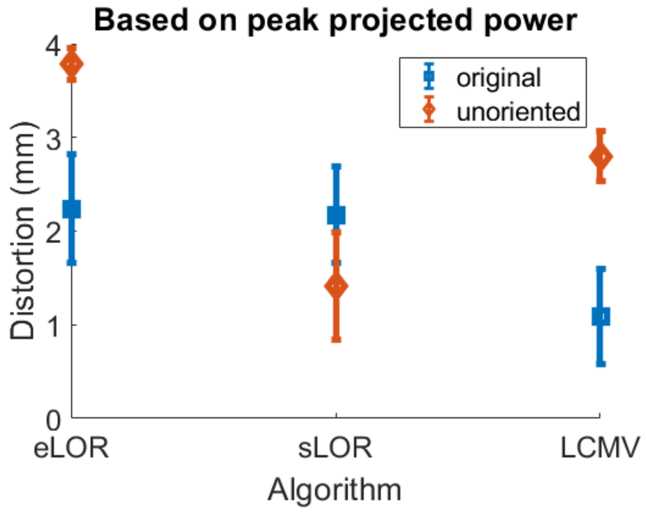

Figure S7. *The use of the normalized projected peak power metric and source orientation constraints. Red diamonds show sources which are allowed to take any orientation; blue squares show sources whose orientation is fixed normal to the cortical mesh. The peak power metric as defined in manuscript equation 12. The algorithms eLOR, sLOR and LCMV correspond to eLORETA (Pascual-Marqui et al., 2011), sLORETA (Pascual-Marqui, 2002) and LCMV((Van Veen et al., 1997). The source consisted of 336 trials of a single dipole (with no spatial extent) simulated at MNI coordinates [x=-40,y= -18,z=48] and a single-trial sensor-level SNR≈18.23dB. Algorithms are implemented in DAiSS. Figure is the output of spm_sim_example_distort_mesh_daiss.m in SPM.*
